# The Surgical Management of an Abdominal Aortic Aneurysm Concomitant With a Renal Tumor Invading the Aortic Wall

**DOI:** 10.7759/cureus.85598

**Published:** 2025-06-09

**Authors:** Yosuke Sugita, Hisashi Sakaguchi, Noriyuki Ito, Keita Yano, Yuki Hori

**Affiliations:** 1 Department of Cardiovascular Surgery, Japan Red Cross Wakayama Medical Center, Wakayama, JPN; 2 Department of Urology, Japan Red Cross Wakayama Medical Center, Wakayama, JPN

**Keywords:** abdominal aortic aneurysm, aortic invasion of malignancy, prognosis, renal neoplasm, two-stage surgery

## Abstract

An abdominal aortic aneurysm (AAA) concomitant with a malignant renal tumor (RMT) invading the aortic wall is an extremely rare and complex condition. The optimal surgical strategy, including procedure type and sequencing, remains unclear. We report the case of a 48-year-old male who presented to our hospital with general fatigue and significant weight loss and was found to have a 58 mm AAA along with a massive left renal tumor adjacent to the aortic wall. Multidisciplinary discussions were held between the cardiovascular surgery and urology teams. The patient underwent a two-stage surgical approach involving open AAA repair using a bifurcated graft, followed by open radical left nephroureterectomy. Histopathological analysis of the renal tumor confirmed urothelial carcinoma. The postoperative course was uneventful, and the patient was discharged nine days after the urological surgery (28 days after the aortic surgery). Two months later, a pulmonary metastasis was detected. Despite multiple lines of chemotherapy, the disease progressed gradually, and the patient died of respiratory impairment caused by rapidly progressive pulmonary metastases 11 months after the initial surgery. The short-term outcome in this patient was favorable; however, more sophisticated treatment strategies should be developed in the future based on further research.

## Introduction

Abdominal aortic aneurysms (AAA) concomitant with renal malignant tumors (RMT) are rare, occurring in approximately 1.6%-4.8% of all AAA cases [1,2]. Among these patients, cases of direct aortic invasion are extremely uncommon, with no previous reports describing the surgical management of this specific condition. Although the European Society for Vascular Surgery (ESVS) recommends a two-stage strategy using endovascular aortic repair (EVAR) for AAA concomitant with an abdominal malignancy without aortic invasion [3], there is no established treatment strategy for patients with aortic invasion. In this report, we present a rare case of AAA concomitant with RMT and aortic invasion and describe its perioperative management and postoperative course.

## Case presentation

**Figure 1:**
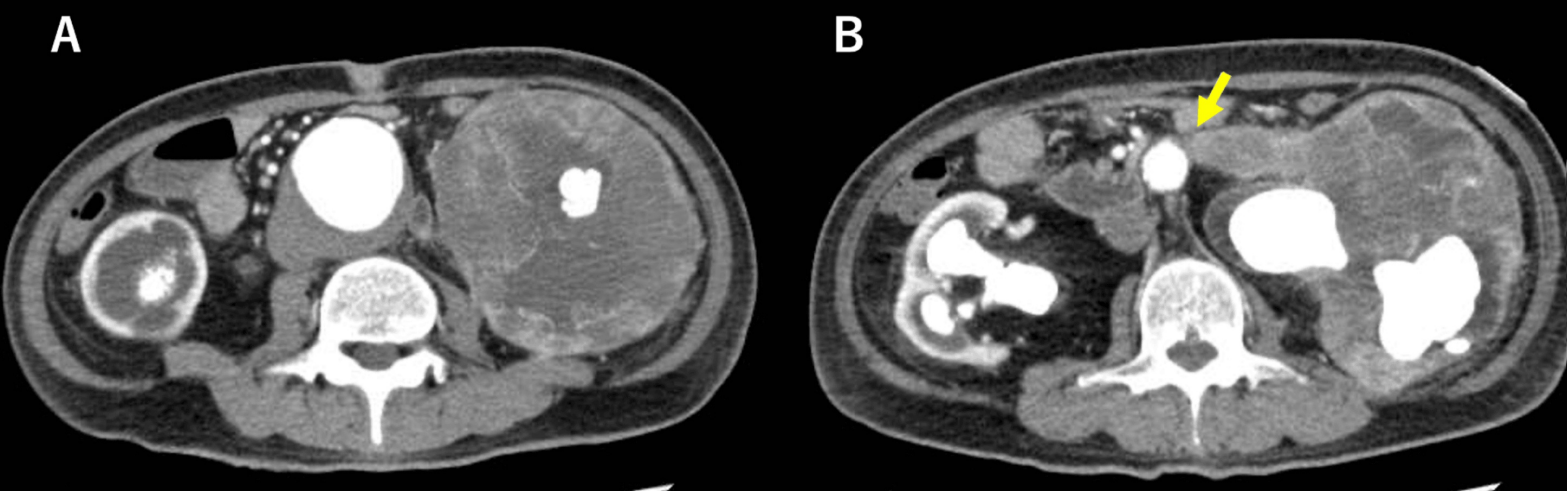
Contrast-enhanced CT A: Giant left RT and infrarenal AAA 58 mm in diameter. B: The RT is adjacent to the aortic wall (arrow) AAA: abdominal aortic aneurysm; CT: computed tomography; RT: renal tumor

A 48-year-old male with no significant medical history visited his primary care physician for fatigue and severe weight loss (9 kg over six months). Blood tests revealed serum creatinine levels as high as 4.0 mg/dL, and the patient was referred to a nephrologist at our hospital. Contrast-enhanced CT revealed a giant left renal tumor with a large staghorn calculus and an AAA measuring 58 mm in diameter (Figure 1A). The left wall of the infrarenal abdominal aorta was adjacent to the renal tumor (Figure 1B).

The cardiovascular surgery and urology teams were consulted, and a multidisciplinary discussion was held to determine the optimal treatment strategy, including the sequencing and management of each pathology. The AAA was deemed suitable for surgical intervention since its diameter exceeded 55 mm. In contrast, the renal mass, which might have represented pyonephrosis from chronic infection, could have been either malignant or benign, and it was unclear whether resection of the mass was necessary. Therefore, histological evaluation was required to determine the appropriate treatment for the renal lesion. Following the multidisciplinary discussion, we planned a two-stage strategy in which the AAA would be treated first, and a histological specimen of the renal tumor would be excised during surgery; we would decide how to treat the renal tumor based on the results of histological evaluation. We chose open surgery rather than EVAR followed by percutaneous renal biopsy, given the patient’s young age and intention for radical treatment of the renal tumor, which was possibly malignant and invading the aortic wall.

**Figure 2:**
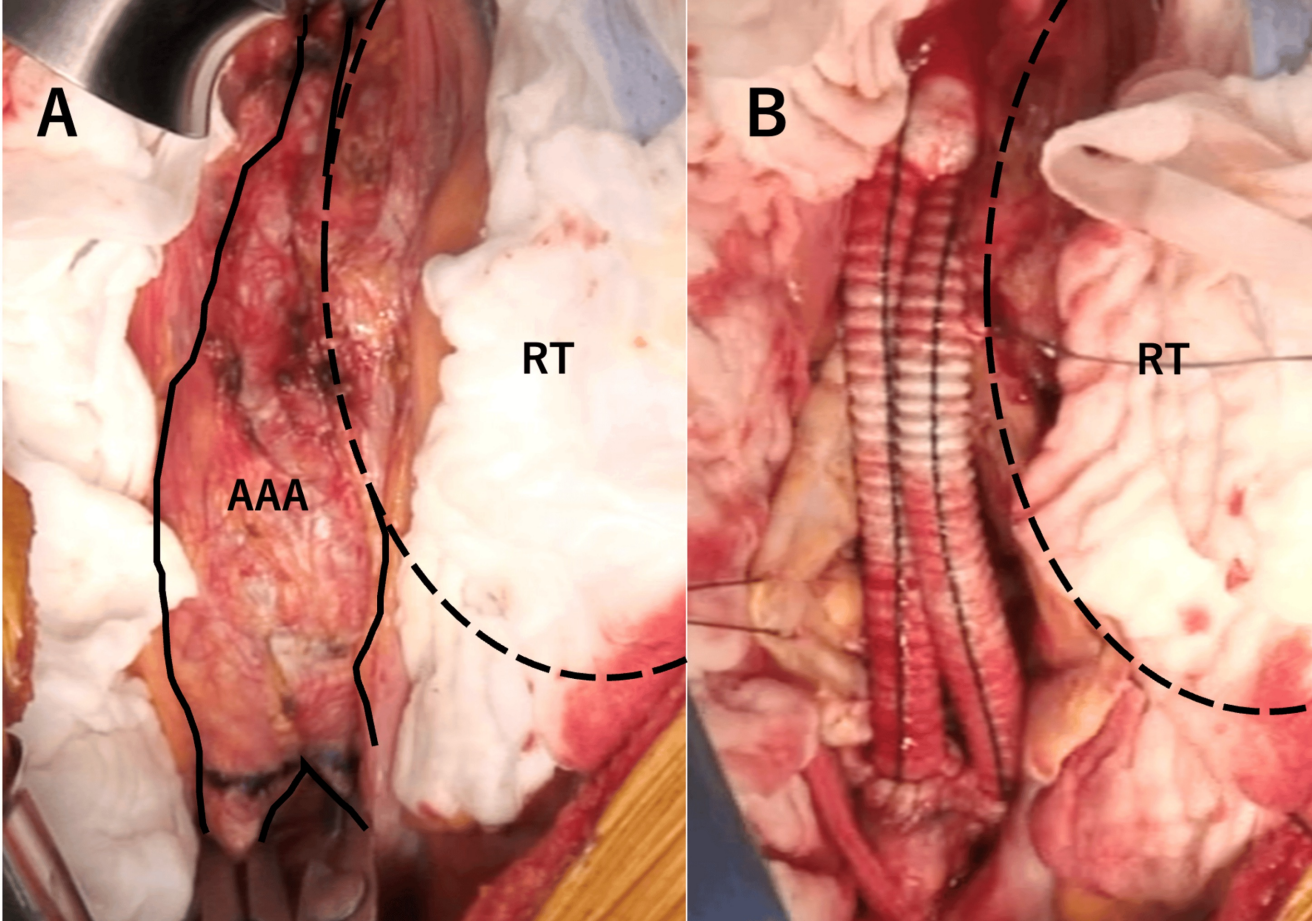
Intraoperative findings A: The left-sided border between the AAA and RT is unclear; only a small region of the infrarenal aorta and bilateral common iliac artery is intact. B: Aortic replacement is performed, leaving the left side of the aorta untouched AAA: abdominal aortic aneurysm; RT: renal tumor

Considering the significant renal impairment and the possibility of perioperative dialysis, an arteriovenous fistula was first created in the patient’s left forearm. Subsequently, open AAA replacement was performed. A midline laparotomy was performed to approach the infrarenal abdominal aorta via the transperitoneal route. In most infrarenal regions of the aorta, the left border of the aortic wall was indistinct because of the adjacent mass. In small areas just under the bifurcation of bilateral renal arteries and around the bilateral common iliac arteries, the vascular structures remained intact and could be encircled (Figure 2A). The AAA was replaced with a 14 × 8 mm Dacron-knitted bifurcated graft (Figure 2B), and a 1 cm² histological specimen of the renal mass was excised. The graft was wrapped in the remaining native aortic wall tissue. After the surgery, the patient was extubated in the operating room and transferred to the ICU. Two days later, the patient developed acute pancreatitis, which was managed after several days of fasting. The patient was discharged from the ICU six days after the operation.

**Figure 3:**
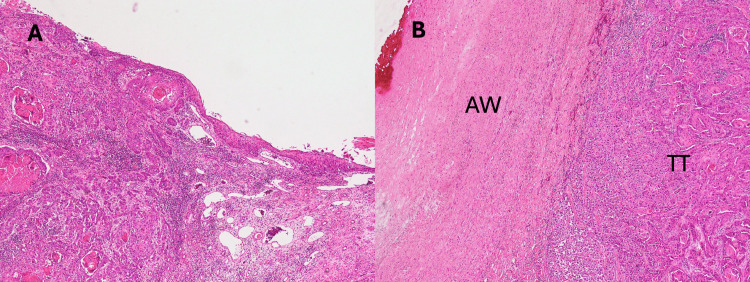
Histological evaluation of the tumor and invaded aortic wall A: Hematoxylin and eosin staining; original magnification ×40. The tumor exhibits characteristics of both urothelial and squamous cell carcinomas. B: Hematoxylin and eosin staining; original magnification: ×40. Tumor tissue invading the aortic wall is observed AW: aortic wall; TT: tumor tissue

Histological examination confirmed a urothelial carcinoma with squamous metaplasia. Preoperative imaging revealed no evidence of metastasis. The preoperative diagnosis was renal pelvic cancer, staged cT3 or cT4 N0 M0. Subsequently, radical nephroureterectomy was performed on postoperative day 19 after AAA surgery. In the urologic operation, a part of the residual aortic wall was resected along with the tumor because of the strong adhesion between the tumor and wall tissue. Pathohistological evaluation of the resected tissues confirmed urothelial carcinoma with squamous differentiation (Figure 3A) and aortic invasion of the tumor (Figure 3B). The pathological stage was pT4, and adjuvant therapy was planned after postoperative recovery due to the incomplete resection of the mass. The patient was discharged without complications on day nine after nephroureterectomy.

Throughout the treatment course, the patient did not develop any complications related to the replaced artificial graft, such as graft infection or thrombosis of the graft limbs. Two months after the effects of surgery subsided, adjuvant therapy with gemcitabine and carboplatin was initiated. However, during the same period, pulmonary metastases were incidentally detected. Due to disease progression, the chemotherapy regimen was switched to pembrolizumab; however, progression could not be controlled. The patient was able to maintain his daily activities until postoperative month 11 when he presented to the emergency department with severe dyspnea caused by progressive pulmonary metastasis; he died on the day of admission.

## Discussion

The management of AAA concomitant with RMT invading the aortic wall can be complex as multiple decisions need to be made, including whether to perform a single- or two-stage operation, and, if a two-stage approach is chosen, which condition should be treated first [1,4]. Additionally, two options are available for AAA treatment: open surgery or EVAR. There is a lack of corroborated evidence to determine which strategy (single- or two-stage) is superior; it is essential to hold multidisciplinary discussions among the relevant departments for each case, and the advantages and disadvantages of each approach should be thoroughly analyzed.

Lewier et al. performed a systematic review [2] of 89 patients with AAA concomitant with renal malignancies; 62 underwent single-stage operations and 27 had two-stage operations. The advantage of the single-stage approach is that AAA and RMT can be treated simultaneously without delay. However, it is extensively surgically invasive, and the operative mortality rate is as high as 3.8%. In contrast, the two-stage approach reduces the surgical burden, reducing physical stress; according to a systematic review, the operative mortality rate of two-stage surgery is 0%. The disadvantages of the two-stage approach are possible adhesions between the target organ and surrounding tissues at the time of the second operation and the risk of progression of the untreated condition during the interval between surgeries.

The organ that should be treated first is also crucial. Some studies recommend that symptomatic pathology be treated first [1,3]. Several studies have investigated the progression of residual pathologies. Choosing AAA repair first has been reported to increase the risk of tumor progression if a long interval period is set. However, whether treating the tumor first is associated with an increased risk of AAA enlargement and rupture remains controversial. Some reports have stated that the rupture risk is as high as 6%-10% within the first three postoperative weeks of tumor resection. In addition, certain reports have highlighted the rapid expansion of AAA during chemotherapy [1,2,5]. However, other reports have indicated that concomitant malignancy and chemotherapy do not affect AAA rupture and expansion [6,7], and the ESVS guidelines conclude that there is no evidence of expansion and rupture risk of AAA concomitant with malignancy; as such, prophylactic AAA repair under a normal diameter threshold is not recommended [3].

The choice between open surgery and EVAR is an important consideration in AAA repair. EVAR is a much less invasive procedure, potentially allowing for a shorter interval between the staged operations [1,2,8]. However, because the aortic wall remains in the body, EVAR may not be sufficiently radical and may be inappropriate in cases of suspected aortic wall invasion. In addition, some reports have noted that graft limb occlusion tends to occur more frequently with EVAR than with open surgical repair [9].

## Conclusions

We discussed a rare case of AAA concomitant with an RMT invading the aortic wall. A two-stage procedure was performed, and the patient had a favorable short-term prognosis. However, the patient died of respiratory impairment caused by pulmonary metastasis 11 months after the initial surgery. It is essential to conduct multidisciplinary discussions between vascular and urology teams for each case, thoroughly examining the surgical approach, sequence of procedures, and histological diagnosis. Exploration of treatment strategies for this lethal condition should continue until best practices are established.
